# Comparison of surgical outcomes of trabeculectomy, Ahmed shunt, and Baerveldt shunt in uveitic glaucoma

**DOI:** 10.1186/s12348-018-0150-y

**Published:** 2018-06-18

**Authors:** Audrey Chow, Bruce Burkemper, Rohit Varma, Damien C. Rodger, Narsing Rao, Grace M. Richter

**Affiliations:** 1 Department of Ophthalmology, USC Roski Eye Institute, Keck Medicine of University of Southern California, 1450 San Pablo Street, Suite 4700, Los Angeles, CA 90033 USA; 2 Department of Ophthalmology, Kaiser Permanente Los Angeles Medical Center, 1515 N. Vermont Ave, 7th floor, Los Angeles, CA 90027 USA

**Keywords:** Glaucoma, Uveitis, Trabeculectomy, Ahmed, Baerveldt, Aqueous shunt

## Abstract

**Background:**

Uveitis is defined as a collection of syndromes involving intraocular inflammation which can lead to pain, tissue damage, and vision loss. Ophthalmic surgery in uveitis patients can be challenging due to inflammation-induced fibrosis and scarring. Trabeculectomy and implantation of glaucoma drainage devices (aqueous shunts) have been used in surgical management of uveitic glaucoma, however there is a paucity of literature examining the comparative results of these entities in this unique setting. The purpose of this retrospective comparative study is to compare clinical outcomes of trabeculectomy with MMC, Ahmed shunt, and Baerveldt shunt surgery specifically in uveitic glaucoma.

**Results:**

Median IOP, IOP reduction, glaucoma medication use, and visual acuity at 6- and 12-month follow-up were similar across groups. Postoperative hypotony rate was significantly different across trabeculectomy (53%), Baerveldt (24%), and Ahmed (18%) groups (*p* = 0.027); other complication rates were similar. Baerveldt eyes had a lower failure rate compared to trabeculectomy (*p* = 0.0054) and Ahmed (*p* = 0.0008) eyes.

**Conclusions:**

While there was no difference in IOP reduction between trabeculectomy, Ahmed, and Baerveldt, Baerveldt eyes had the lowest failure rate.

## Background

Uveitis is defined as a collection of syndromes involving intraocular inflammation which can lead to pain, tissue damage, and vision loss [1, 2]. With an estimated 109,000 cases of uveitis in the USA and 43,000 new cases a year, it is the third leading cause of preventable blindness in the developed world [3]. Twenty percent of patients with uveitis develop glaucoma due to inflammation or secondary to corticosteroid treatment of uveitis [2–4]. In uveitic glaucoma, elevated intraocular pressure results from increased resistance to aqueous outflow and can lead to irreversible optic nerve damage and eventual blindness [3].

Ophthalmic surgery in uveitis patients can be challenging due to inflammation-induced fibrosis and scarring. However, surgical intervention may be indicated if intraocular pressure (IOP) cannot be adequately controlled by medications alone. These procedures include trabeculectomy and implantation of glaucoma drainage devices (aqueous shunts). Two types of aqueous shunts are the valved Ahmed shunt and the non-valved Baerveldt shunt [5]. Previous studies have separately demonstrated that trabeculectomy, Ahmed shunt, and Baerveldt shunt are all reasonably effective in controlling uveitic glaucoma [6–9]. Prior literature suggests that while trabeculectomy has good outcomes in uveitic glaucoma in the short term, the risk of failure is relatively high in the long term [6, 10–12]. Bettis et al. compared the outcomes of trabeculectomy with mitomycin C (MMC) and Ahmed valve implantation and found that the Ahmed device was more effective than the trabeculectomy in uveitic eyes [13]. Iverson et al. showed that the non-valved Baerveldt device was also likely better than the trabeculectomy with MMC in controlling uveitic glaucoma [14]. Between the Ahmed and Baerveldt shunts, both the Ahmed Baerveldt Comparison Study (ABC) and the Ahmed Versus Baerveldt Study (AVB) found that Baerveldt patients had lower failure rates and lower median postoperative IOP but experienced more early postoperative complications, namely, hypotony [15–18]. However, these studies examined patients with glaucoma of a broad range of etiologies, not uveitic glaucoma specifically. Hypotony is especially relevant to uveitis where there is an increased risk of postoperative hypotony due to ciliary body inflammation and shutdown [4]. The Ahmed valve relies on the Venturi effect to open the valve for fluid outflow by facilitating a pressure differential when the pressure within the eye reaches an upper threshold (8–12 mm). The tension on the membranes within the valve is responsible for reducing hypotony due to overfiltration [19–21]. In the setting of low aqueous production, theoretically, the Ahmed valve has this additional mechanism in place to reduce hypotony, whereas the Baerveldt does not. While the risk of hypotony due to overfiltration would be lower with the Ahmed, there would still be hypotony if there were significant aqueous underproduction.

Direct comparison of outcomes of trabeculectomy with MMC, Ahmed, and Baerveldt surgery in uveitic patients is yet to be reported. The purpose of this retrospective comparative study is to compare clinical outcomes of these entities specifically in the setting of uveitic glaucoma. We hypothesized that trabeculectomies would have poorer outcomes than aqueous shunts due to persistent inflammation and postoperative scarring. Additionally, considering the increased risk hypotony in uveitic patients, we predicted that the valved Ahmed shunt would have lower rates of hypotony than the non-valved Baerveldt shunts in patients with uveitic glaucoma.

## Results and discussion

A total of 147 eyes from 147 patients that met the study criteria were identified and reviewed. Seventeen eyes (17 patients) underwent trabeculectomy with MMC, 22 eyes (22 patients) underwent Ahmed implantation, and 108 eyes (108 patients) underwent Baerveldt implantation. One trabeculectomy, 11 Baerveldt, and 2 Ahmed eyes also underwent combined procedures with cataract extraction with intraocular lens implant at the time of glaucoma surgery. A total of 15 eyes underwent cataract surgery during the follow-up period.

Patient baseline demographics and ocular characteristics are summarized in Table 1. There were no significant differences in these entities across groups. The median age of the study population was 62 years (19–96); 57% of the study population were female, and 14% were diabetic. The median IOP before surgery was 35 mmHg (19–85) on four (0–5) topical glaucoma medications and one (0–3) topical anti-inflammatory medications. Forty-eight percent of patients were on systemic glaucoma medications, and 14% were on immunosuppressive medications. The median visual acuity was 0.7 (0–3) logMAR units. 110/147 (75%) of patients had prior eye surgery, 22 of which had prior glaucoma filtering surgery. Fifty-nine percent eyes were pseudophakic with no significant difference in the proportion of pseudophakia across groups (*p* = 0.96). Fifty-eight percent of the eyes had idiopathic uveitis. The most common uveitis location in all groups was anterior uveitis (*n* = 127), and the most common known etiologies were herpes simplex virus (*n* = 18), uveitis-glaucoma-hyphema syndrome (*n* = 9), and Vogt-Koyanagi syndrome (*n* = 6). Other etiologies included sarcoidosis, birdshot, ankylosing spondylitis, juvenile rheumatoid arthritis, rheumatoid arthritis, syphilis, Posner-Schlossman syndrome, Fuch’s heterochromic iridocyclitis, parasite, human leukocyte antigen B27, and tuberculosis.

**Table 1:** Baseline demographics and ocular characteristics

	Total	Trabeculectomy	Ahmed	Baerveldt	*p* value
	*n* = 147 eyes	*n* = 17 eyes	*n* = 22 eyes	*n* = 108 eyes	
Age (years), M (range)	62 (19–96)	54 (19–72)	61 (22–86)	62 (20–96)	0.15*
Sex, female, % (*n*)	57 (84)	59 (10)	50 (11)	58 (63)	0.70^§^
Diabetes, % (*n*)	14 (20)	6 (1)	9 (2)	16 (17)	0.57^†^
Preop IOP (mmHg), M (range)	35 (19–85)	34 (23–50)	40 (26–58)	33 (19–85)	0.066*
Preop visual acuity (logMAR), M (range)	0.7 (0–3)	0.5 (0–3)	0.6 (0–3)	0.8 (0–3)	0.34*
Preop number of topical glaucoma medications, M (range)	4 (0–5)	4 (0–1)	3 (0–4)	4 (0–5)	0.15*
Preop on systemic glaucoma medications, % (*n*)	48 (70)	53 (9)	55 (12)	45 (49)	0.66^†^
Preop number of topical anti-inflammatory medications, M (range)	1 (0–3)	1 (0–2)	1 (0–2)	1 (0–3)	0.82*
Preop on systemic immunosuppressants, % (*n*)	14 (21)	18 (3)	18 (4)	13 (14)	0.63^†^
Prior eye surgery, % (*n*)	75 (110)	82 (14)	75 (81)	68 (15)	0.60^§^
Prior glaucoma surgery, % (*n*)	15 (22)	24 (4)	14 (3)	14 (15)	0.22^§^
Pseudophakia, % (*n*)	56 (82)	53 (9)	55 (12)	56 (61)	0.96^†^
Uveitis location, *n* (%)					
Anterior	127 (86)	13 (76)	20 (90)	94 (87)	
Intermediate	4 (3)	1 (6)	0 (0)	3 (3)	
Posterior	4 (3)	2 (12)	1 (5)	1 (0.9)	
Pan	12 (8)	1 (6)	1 (5)	10 (9)	
Etiology, *n* (%)					
Idiopathic	91 (62)	12 (71)	11 (50)	69 (64)	
Herpes simplex virus	18 (12)	2 (12)	1 (5)	15 (14)	
Sarcoidosis	1 (0.7)	0 (0)	0 (0)	1 (0.9)	
Birdshot	1 (0.7)	0 (0)	1 (5)	0 (0)	
Ankylosing spondylitis	2 (1)	0 (0)	2 (9)	0 (0)	
Vogt-Koyanagi syndrome	6 (4)	1 (6)	2 (9)	3 (3)	
Juvenile rheumatoid arthritis	3 (2)	1 (6)	1 (5)	1 (0.9)	
Uveitis-glaucoma-hyphema syndrome	9 (6)	0 (0)	1 (5)	8 (7)	
Rheumatoid arthritis	5 (3)	1 (6)	0 (0)	4 (4)	
Syphilis	1 (0.7)	0 (0)	1 (5)	0 (0)	
Posner-Schlossman syndrome	1 (0.7)	0 (0)	0 (0)	1 (0.9)	
Fuch’s heterochromic iridocyclitis	1 (0.7)	0 (0)	0 (0)	1 (0.9)	
Parasite	2 (1)	0 (0)	2 (9)	0 (0)	
HLA B27	4 (3)	0 (0)	0 (0)	4 (4)	
Tuberculosis	1 (0.7)	0 (0)	0 (0)	1 (0.9)	

*IOP* intraocular pressure, *logMAR* logarithm of minimum angle of resolution, *HLA* human leukocyte antigen

*Kruskal-Wallis test; ^†^Fisher’s exact test; ^§^chi-squared test

As shown in Table 2, at 6-month follow-up, median IOP was reduced from 34 (23–50) to 10 (1–25) mmHg in the trabeculectomy group, 40 (26–58) to 16 (8–28) mmHg in the Ahmed group, and 33 (19–85) to 12 (5–33) mmHg in the Baerveldt group (*p* = 0.015). Further, pairwise comparison showed that Ahmed IOP at 6 months was significantly higher than that of trabeculectomy (*p* = 0.02) and of Baerveldt (*p* = 0.0086), while there was no significant difference between trabeculectomy and Baerveldt (*p* = 0.32). Both the point and percent IOP reduction in trabeculectomy (22 (11–47) mmHg, 69% (41–97)), Ahmed (24 (7–40) mmHg, 65% (21–81)), and Baerveldt (21 (5–75) mmHg, 64% (40–89)) groups were similar (*p* = 0.56). The median number of postoperative topical glaucoma medications was 0 (1–3) in the trabeculectomy group, 2 (0–4) in the Ahmed group, and 2 (0–4) in the Baerveldt group (*p* = 0.098). The percentage of patients on systemic glaucoma medications postoperatively was 6% in the trabeculectomy group, 9% in the Ahmed group, and 6.5% in the Baerveldt group (*p* = 0.86). Topical glaucoma, anti-inflammatory, systemic glaucoma, immunosuppressive medication use, change in visual acuity, and rate of follow-up cataract surgery were similar in all groups.

**Table 2:** Six-month postoperative outcome

	Total	Trabeculectomy	Ahmed	Baerveldt	*p* value
	*n* = 147 eyes	*n* = 17 eyes	*n* = 22 eyes	*n* = 108 eyes	
IOP (mmHg), M (range)	12 (1–33)	10 (1–25)	16 (8–28)	12 (5–33)	0.015*
Visual acuity (logMAR), M (range)	0.7 (0–3)	0.4 (0–3)	1 (0–3)	0.7 (0–3)	0.14*
Number of topical glaucoma medications, M (range)	2 (0–4)	0 (0–3)	2 (0–4)	2 (0–4)	0.098*
On systemic glaucoma medications, % (*n*)	7 (10)	6 (1)	9 (2)	6.5 (7)	0.86^†^
Number of topical anti-inflammatory medications, M (range)	1 (0–3)	1 (0–2)	1 (0–2)	1 (0–3)	0.77*
On systemic immunosuppressants, % (*n*)	10 (14)	6 (1)	2 (3)	9 (10)	0.65^§^
IOP reduction (mmHg), M (range)	22 (5–75)	22 (11–47)	24 (7–40)	21 (5–75)	0.56*
% IOP reduction, M (range)	65 (26–100)	69 (41–97)	65 (21–81)	64 (40–89)	0.43*
Change in visual acuity (logMar), M (range)	0 (− 3–2)	− 0.1 (− 1–2)	0 (− 2–2)	0 (− 3–2)	0.52*
Lost > 2 lines in visual acuity, % (*n*)	25 (36)	6 (1)	31 (7)	26 (28)	0.14^§^
Cataract surgery during follow up period, % (*n*)	5 (7)	0 (0)	5 (1)	6 (6)	1^†^
Failure, % (*n*)	4 (6)	12 (2)	14 (3)	0.9 (1)	0.0063^†^

Failure was defined as postoperative intraocular pressure greater than 21 mmHg or less than 5 mmHg, < 20% IOP reduction, reoperation, or loss of light perception

*IOP* intraocular pressure, *logMAR* logarithm of minimum angle of resolution

*Kruskal-Wallis test; ^†^Fisher’s exact test; ^§^chi-squared test

Outcome measures at 12-month follow-up are shown in Table 3. At that time point, there was no significant difference in absolute IOP (*p* = 0.19), point IOP reduction (*p* = 0.62), or percent IOP reduction (*p* = 0.76) across groups. Median IOP, point IOP reduction, and percent IOP were 10 (5–38) mmHg, 22 (10–39) mmHg, and 69 (36–82)%, respectively, in the trabeculectomy group; 14 (6–27) mmHg, 24 (11–46) mmHg, 67 (29–86)%, respectively, in the Ahmed group; and 12 (4–38) mmHg, 21 (8–76) mmHg, and 65 (38–89)%,respectively, in the Baerveldt group. The median number of postoperative topical glaucoma medications was 0 (0–4) in the trabeculectomy group, 2 (0–4) in the Ahmed group, and 2 (0–4) in the Baerveldt group (*p* = 0.22). The percentage of patients on systemic glaucoma medications postoperatively was 9% in the trabeculectomy group, 6% in the Ahmed group, and 4% in the Baerveldt group (*p* = 0.86). Again, there was also no difference in topical glaucoma, topical anti-inflammatory, systemic glaucoma, immunosuppressive medication use, change in visual acuity, or rate of follow-up cataract surgery between groups.

**Table 3:** Twelve-month postoperative outcome

	Total	Trabeculectomy	Ahmed	Baerveldt	*p* value
	*n* = 115 eyes	*n* = 11 eyes	*n* = 17 eyes	*n* = 87 eyes	
IOP (mmHg), M (range)	12 (4–38)	10 (5–38)	14 (6–27)	12 (4–38)	0.19*
Visual acuity (logMAR) M (range)	0.6 (0–3)	0.2 (0–1)	0.5 (0–3)	0.7 (0–3)	0.097*
Number of topical glaucoma medications, M (range)	2 (0–4)	0 (0–4)	2 (0–4)	2 (0–4)	0.22*
On systemic glaucoma medications, % (*n*)	5 (6)	9 (1)	6 (1)	4 (4)	0.59^†^
Number of topical anti-inflammatory medications, M (range)	1 (0–2)	1 (0–2)	1 (0–2)	1 (0–2)	0.69*
On systemic immunosuppressants, % (*n*)	5 (6)	9 (1)	6 (1)	4 (4)	0.59^†^
IOP reduction (mmHg), M (range)	22 (10–76)	22 (10–39)	24 (11–46)	21 (8–76)	0.62*
% IOP reduction, M (range)	66 (− 38–89)	69 (36–82)	67 (29–86)	65 (38–89)	0.76*
Change in visual acuity (logMar), M (range)	0 (− 3–2)	− 0.3 (− 1–0.2)	0 (−1–2)	0 (− 3–2)	0.48*
Lost > 2 lines in visual acuity, % (*n*)	33 (38)	36 (4)	41 (7)	31 (27)	0.70^§^
Cataract surgery during follow up period, % (*n*)	13 (15)	0 (0)	6 (1)	16 (14)	0.35^†^
Failure, % (*n*)	7.5 (11)	18 (3)	23 (5)	3 (3)	0.0015^†^

Further pairwise comparison showed a significant difference in 6 months IOP in Ahmed versus trabeculectomy (*p* = 0.02) and Ahmed versus Baerveldt (*p* = 0.0086). There was no significant difference in 6 months IOP between trabeculectomy versus Baerveldt (*p* = 0.32)

Failure was defined as postoperative intraocular pressure greater than 21 mmHg or less than 5 mmHg, < 20% IOP reduction, reoperation, or loss of light perception

*IOP* intraocular pressure, *logMAR* logarithm of minimum angle of resolution

*Kruskal-Wallis test; ^†^Fisher’s exact test; ^§^chi-squared test

The type, rate, time, and total number of postoperative complications were similar in all groups (*p* > 0.05), except for hypotony (Tables 4 and 5). Specifically, there was a significant difference in early (6 weeks to 4 months postop) hypotony rate in trabeculectomy (47%), Ahmed (18%), and Baerveldt eyes (18.5%) (*p* = 0.027) but not for late (> 4 months postop) hypotony rate (trabeculectomy 6%, Ahmed 0%, Baerveldt 7%) (*p* = 0.53).

**Table 4:** Early postoperative complications

Complication, % (*n*)	Total	Trabeculectomy	Ahmed	Baerveldt	*p* value
	*n* = 147 eyes	*n* = 17 eyes	*n* = 22 eyes	*n* = 108 eyes	
Hypotony	22 (32)	47 (8)	18 (4)	19 (20)	0.027
Choroidal effusion	11 (16)	0 (0)	9 (2)	13 (14)	0.34
Cystoid macular edema	5 (8)	6 (1)	0 (0)	7 (7)	0.70
Hyphema	8 (11)	12 (2)	9 (2)	7 (7)	0.59
Shallow anterior chamber	3 (5)	6 (1)	0 (0)	4 (4)	0.57
Corneal edema/decompensation	17 (25)	6 (1)	9 (2)	20 (22)	0.26
Uveitic flare	9 (13)	0 (0)	4.5 (1)	11 (12)	0.44
Diplopia/strabismus	0.7 (1)	0 (0)	0 (0)	0.9 (1)	1
Blocked tube	0.7 (1)		0 (0)	0.9 (1)	1
Erosion	2 (3)		5 (1)	2 (2)	0.43
Revision	8 (12)	6 (1)	5 (1)	9 (10)	0.87
Patients with complications	69 (101)	65 (11)	68 (15)	69 (75)	0.92
Total number of complications	2 (220)	1 (21)	1 (27)	2 (172)	0.26

Early postoperative complications were defined as complications occurring within 6 weeks to 4 months after surgery. The Fisher’s exact test was used for statistical analysis

**Table 5:** Late postoperative complications

Complication, % (*n*)	Total	Trabeculectomy	Ahmed	Baerveldt	*p* value
	*n* = 147 eyes	*n* = 17 eyes	*n* = 22 eyes	*n* = 108 eyes	
Hypotony	6 (9)	6 (1)	0 (0)	7 (8)	0.53
Choroidal effusion	1 (2)	0 (0)	5 (1)	0.9 (1)	0.46
Cystoid macular edema	6 (9)	6 (1)	5 (1)	7 (8)	0.14
Hyphema	1 (2)	0 (0)	0 (0)	2 (2)	1
Shallow anterior chamber	0 (0)	0 (0)	0 (0)	0 (0)	1
Corneal edema/decompensation	12 (18)	6 (1)	5 (1)	15 (16)	0.41
Uveitic flare	0.7 (1)	0 (0)	0 (0)	0.9 (1)	1
Diplopia/strabismus	1 (2)	0 (0)	5 (1)	0.9 (1)	0.46
Blocked tube	2 (3)		0 (0)	3 (3)	1
Erosion	3 (5)		9 (2)	3 (3)	0.20
Revision	9 (13)	0 (0)	24 (5)	8 (9)	0.055
Patients with complications	38 (56)	24 (4)	46 (10)	39 (42)	0.36
Total number of complications	0.8 (110)	0.4 (6)	0.8 (17)	0.8 (87)	0.12

Late postoperative complications were defined as complications occurring > 4 months after surgery. The Fisher’s exact test was used for statistical analysis

Overall, the most common cause of failure was the failure of IOP control (*n* = 14); three trabeculectomy, five Ahmed, and five Baerveldt eyes failed due to IOP > 21 mmHg, and one Baerveldt eye failed due to persistent hypotony (defined as IOP < 5 mmHg). Two Baerveldt eyes lost light perception. One Ahmed was removed at a patient’s request due to tube dysesthesia, and one Baerveldt was removed due to tube exposure. One patient received a possibly defective Ahmed with no bleb formation.

**Fig. 1:**
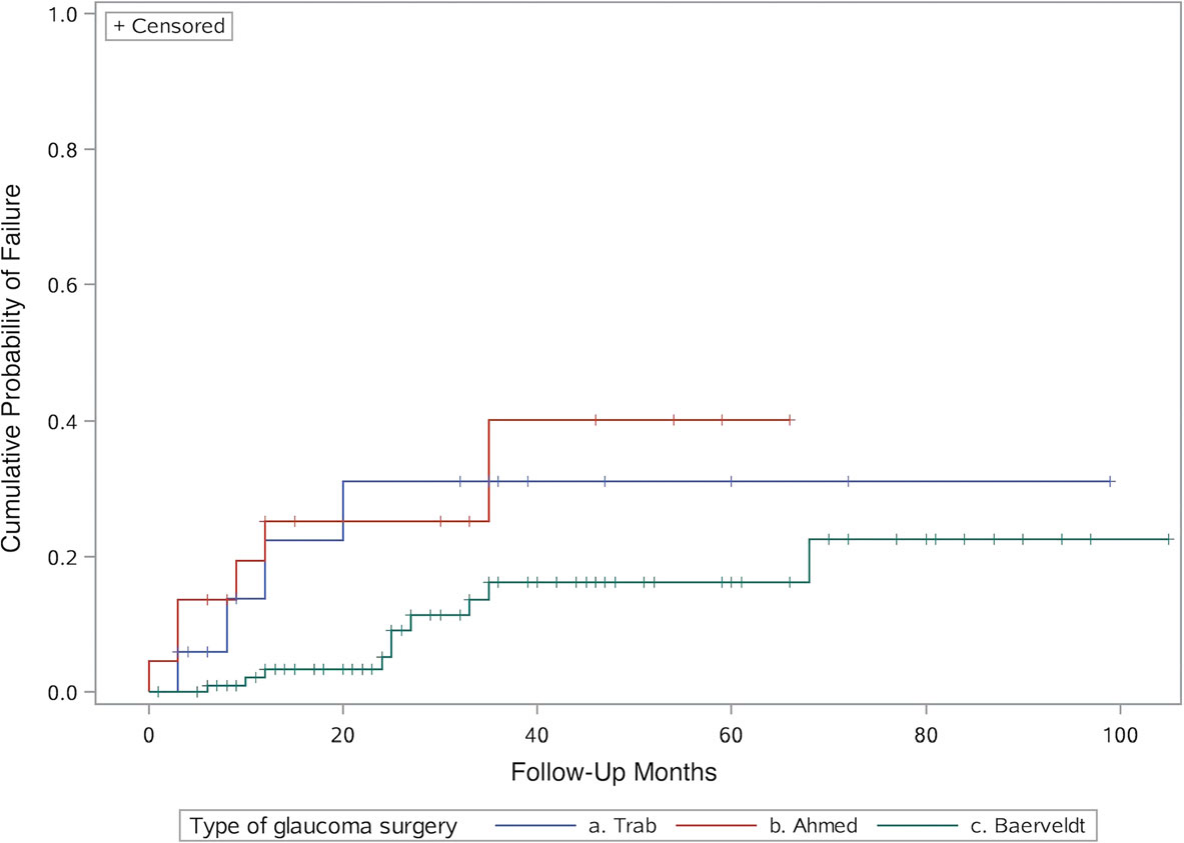
Cumulative probability of failure from any cause. Kaplan-Meier curves showing cumulative probability of failure from any cause. Ahmed and trabeculectomy were similar (*p* = 0.93, Wilcoxon test). Baerveldt differed significantly from trabeculectomy (*p* = 0.0054, Wilcoxon test) and from Ahmed (*p* = 0.0008, Wilcoxon test)

At 6-month follow-up, 0.9% of Baerveldt eyes had failed, followed by 12% of trabeculectomy eyes and 14% of Ahmed eyes (*p* = 0.0063) (Table 2). This trend persisted through 12 months of follow-up, with Baerveldts at 3%, trabeculectomy at 18%, and Ahmed at 23% (*p* = 0.0015) failure rate (Table 3). Figure 1 illustrates the cumulative probability of failure in each group using Kaplan-Meier survival analysis. While the cumulative probability of failure was similar between the trabeculectomy and Ahmed groups (*p* = 0.93), the Baerveldt group had a significantly lower cumulative probability of failure than both the trabeculectomy (*p* = 0.0054, Wilcoxon test) and the Ahmed (*p* = 0.0008, Wilcoxon test) groups (Table 6).

**Table 6:** Cumulative failure analysis

	*p* value
Ahmed vs. trabeculectomy	0.93
Baerveldt vs trabeculectomy	0.0054
Baerveldt vs. Ahmed	0.0008

The Wilcoxon test was used for statistical analysis

Univariate analyses were conducted to evaluate baseline demographic and ocular characteristics as possible risk factors for failure (Table 7). Consistent with our other findings, surgery type was a significant predictor of failure (*p* = 0.028). All other possible risk factors were not statistically significant (*p* > 0.05).

**Table 7:** Univariate predictors of failure

Variable	*p* value
Sex	0.057^§^
Age	0.14*
Race	0.25^†^
Smoking	0.70^†^
Alcohol	0.39*
Diabetes	0.48*
Family history of glaucoma	0.91^§^
Pseudophakia	0.14^§^
History of eye surgery	0.26^§^
History of Glc filtration surgery	0.56^§^
History of failed trabeculectomy	0.71^†^
Prednisone	0.68^§^
Uveitis location	0.81^†^
Surgery type	0.028^†^
Preoperative IOP	0.12^§^
Cataract surgery in follow-up period	0.43^†^

*Glc* glaucoma, *IOP* intraocular pressure

*Kruskal-Wallis test; ^†^Fisher’s exact test; ^§^chi-squared test

Table 8 describes the relative rates of failure between each group using logistic regression. Baerveldt eyes were 67% less likely than trabeculectomy eyes to fail (odds ratio 0.33, 95% CI 0.09–0.96) and 73% less likely than Ahmed eyes to fail (odds ratio 0.27, 95% CI 0.09–0.85) overall. The odds ratio for Ahmed versus trabeculectomy failure was 1.22 (95% CI 0.28–5.26).

**Table 8:** Odds ratio of failure

Comparison	Odds ratio	95% confidence interval
Ahmed vs. trabeculectomy	1.22	0.28–5.26
Baerveldt vs trabeculectomy	0.33	0.09–0.96
Baerveldt vs. Ahmed	0.27	0.09–0.85

This study comparing surgical outcomes of trabeculectomy, Ahmed shunt, and Baerveldt shunt in the eyes with uveitic glaucoma showed overall similar IOP control, medication use, and complication rates.

We found that all three treatments appear to be reasonably effective at controlling IOP to similar extents in the eyes with uveitic glaucoma, as previously reported [6–9]. At 12 months, IOP was comparable across groups, with no significant difference found. We observed a comparable decrease in glaucoma, anti-inflammatory, or immunosuppressant medication use in all three groups, with no significant difference in the degree of this reduction between groups. Notably, anti-inflammatory and immunosuppressant use did not increase in any group, demonstrating that these surgical treatment modalities at least did not worsen the uveitis.

The Ahmed group had significantly worse IOP control at 6 months follow-up compared to both trabeculectomy and Baerveldt groups. Previous studies have found that Ahmed shunts experience a higher incidence of hypertensive phase (IOP > 21 mmHg) that can last from 3 weeks to several months after surgery [22]. This hypertensive phase could potentially extend into the 6-month follow-up period, contributing to the higher 6-month IOP in Ahmed eyes found in our study. One theory for this observation is that immediate filtration causes an influx of inflammatory factors contained in the aqueous fluid; this stimulates a fibrotic response in the subconjunctival space, leading to bleb encapsulation [23]. It is probable that this fibrotic response may be exaggerated in uveitic glaucoma due to the inflammatory nature of uveitis. Notably, both the trabeculectomy and Baerveldt group also experienced greater rates of early hypotony compared to the Ahmed group, which could have additionally contributed to the disparity in IOP reduction. By 12 months, there was no longer a difference in IOP control between the three groups.

In the early postoperative phase specifically, trabeculectomy eyes had the highest rate of hypotony, followed by Baerveldt then Ahmed eyes. This is consistent with the results of the several previous large prospective randomized clinical trials. The Trabeculectomy Versus Tube study (TVT) comparing trabeculectomy versus Baerveldt shunt similarly found significantly higher rates of hypotony with trabeculectomy over Baerveldt shunt [24]. However, the TVT excluded uveitic eyes. With respect to Ahmed shunts, both the ABC study which included 18 uveitic eyes (*n* = 276) and AVB study which included 23 uveitic eyes (*n* = 238) observed greater risk of hypotony in Baerveldt over Ahmed eyes [18, 25, 26].

Interestingly, while 4.5% of Ahmed eyes and 11% of Baerveldt eyes experienced uveitic flares during the early or late postoperative period, there were no instances of this in trabeculectomy eyes. This may well be due to sampling bias, as uveitic eyes with greater inflammation at baseline may be seen as poor candidates for trabeculectomy, and thus are more likely to receive a shunt. Alternatively, this difference may be due to aqueous shunt implants inducing more inflammation than trabeculectomy with MMC. The placement of a foreign material like silicone may incite an inflammatory response; silicone has a high affinity for plasma proteins and a tendency to activate inflammatory mediators as well as stimulate fibrosis [27, 28]. This would be particularly unfavorable in uveitic patients who are already predisposed to severe inflammation.

Notably, both shunts tended to experience more corneal decompensation than trabeculectomy. Corneal complications associated with shunts have been observed in previous studies [27, 29]. In our study, Baerveldt eyes trended towards greater rates of corneal complications compared to Ahmed eyes, a finding that was significant in the ABC and ABV studies [15–18]. One case report observed backflow of aqueous fluid toward the cornea in a Baerveldt shunt, a possible mechanism of corneal damage [28].

Overall, Baervelt eyes appear to experience more complications than the other treatment groups. Specifically, both the ABC and ABV studies found more complications associated with Baerveldt than Ahmed eyes [15–18]. Iverson et al. found similar complication rates between Baerveldts and trabeculectomies specifically in uveitic eyes, which echoed the findings in the TVT study. However, the TVT study was a relatively smaller study (*n* = 76), and it did not include uveitic eyes [14, 24]. In our study, we observed a pattern of Baerveldt eyes experiencing greater early and late number of complications, with a trend towards significance. They tended to have more persistent hypotony in the late postoperative period. This could be related to the lack of a valve along with the larger drainage plate size of Baerveldts, leading to overfiltration. However, there have been mixed results on whether a robust correlation between end plate size and IOP reduction exists [22, 30–33]. While we were concerned that this tendency for hypotony with Baerveldt shunts coupled with postoperative ciliary body shutdown in uveitic glaucoma would produce worse outcomes, the vast majority of failures overall were still due to high IOP; only one failure was attributed to persistent hypotony, which was in a Baerveldt eye.

While Baerveldt eyes tended to experience more complications, they ultimately had the lowest failure rate at 6- and 12-month follow-up as well as the lowest overall cumulative probability of failure (Tables 2 and 3, Fig. 1). While the rate of failure found in previous studies has been widely variable, the Baerveldt failure rates have consistently been lower than that of trabeculectomy and Ahmed within each prior study [14–18, 24]. Specifically, at 12-month follow-up, we found failure rates of 3, 18, and 23% (*p* = 0.0015) in Baerveldt, trabeculectomy, and Ahmed eyes, respectively.

While Bettis et al. observed better cumulative success rate with Ahmed shunts than trabeculectomies in uveitic eyes, we did not find a significant difference between these entities [13]. Possible factors for the difference in results between studies include relatively small sample size (*n* = 22) in our study and trend towards higher baseline preoperative IOP in the Ahmed group.

One eye in this group received a possibly defective Ahmed device, as a bleb never formed and IOP remained high despite a clear tube. This patient was also allergic to most glaucoma medications and was less able to control IOP. These factors may have disproportionately negatively affected the overall results of the Ahmed group given the relatively small group size (*n* = 22) in this study.

With regard to IOP, consistent with AVB and ABC studies, Ahmed eyes had higher postoperative pressures at both 6-month (16 (8–28) mmHg, *p* = 0.015) and 12-month (14 (6–27) mmHg, *p* = 0.19) follow-up. However, they also trended towards the greatest IOP point and percent reduction. This suggests that the Ahmed valves were at least as effective as the other treatments at reducing IOP.

It is possible that physicians may be more likely to place Ahmed shunts in patients with higher IOPs that need immediate relief because trabeculectomy may drop the IOP too quickly, while the Baerveldt shunt with tubal ligation would take up to 6 weeks for the sutures to dissolve and open. It could be speculated that patients with severe IOP elevation may benefit most from an Ahmed shunt implant.

This study has potential limitations associated with its retrospective design. These weaknesses include possible selection bias, inconsistent follow-up, and lack of randomization to treatment groups. Fortunately, baseline demographics were similar between our treatment groups. The Kaplan-Meier analysis was used to compare failure rates despite unequal follow-up. The vast majority of our patients had anterior uveitis. While we are unable to comment on any differential effects on anatomical uveitic classifications or uveitic etiologies, this would be a topic of interest for future research. We also had a relatively large disparity in sample sizes between treatment groups. Relatively small trabeculectomy and Ahmed group sizes (particularly trabeculectomy) compared to Baerveldt group size limit the power of our statistical analyses, including that of the pairwise comparisons and logistic regression. Additional eyes in these groups to increase and equalize sample sizes may have better powered our results.

## Conclusions

In summary, we found no difference in median IOP reduction or medication use at 6- and 12-month follow-up between trabeculectomy with MMC, Ahmed shunt, and Baerveldt shunt in patients with uveitic glaucoma. Complication rates were similar with the exception of hypotony; the trabeculectomy group had a higher rate of early hypotony, while the Baerveldt group trended towards a higher rate of late hypotony. Baerveldt eyes had significantly lower failure rates at 6-and 12-month follow-up, as well as lowest overall cumulative failure rate. Prospective, randomized controlled trials are needed to further explore the comparative effects of these surgical treatments in the eyes with uveitic glaucoma.

## Methods

We retrospectively reviewed the records of 147 eyes (17 trabeculectomy with MMC, 22 Ahmed, 108 Baerveldt) from 147 patients who underwent ophthalmic surgery for uveitic glaucoma at the University of Southern California Department of Ophthalmology between 2005 and 2014. Exclusion criteria included patients with < 6 months of follow up. This study was reviewed and approved by the Institutional Review Board at the University of Southern California.

All procedures were performed in a standard fashion, as briefly described below. For all groups, the eyes received subconjunctival injections of cefazolin and dexamethasone and topical steroid-antibiotic ointment before being patched at the end of the procedure.

For trabeculectomy, a fornix-based conjunctival flap was created in the superior quadrant. Next, a 4 × 3-mm trapezoidal half scleral thickness flap was made. An MMC 0.5 mg/mL soaked sponge was then applied under the conjunctival flap for 2 min, which was irrigated copiously with balanced salt solution afterwards. A Kelly punch was used to create a sclerostomy at the limbus. The corners of the scleral flap were then sutured down, and the overlying Tenon and conjunctival tissues were re-approximated and closed.

For the Ahmed placement, a conjunctival periotomy was created. An Ahmed FP-7 implant was placed 8 mm posterior to the limbus in either in the superonasal or superotemporal quadrants and anchored to the sclera with two 8-0 nylon sutures. The tube was trimmed to an appropriate length with a beveled tip. A sclerostomy was made, and the tube is inserted into the anterior chamber through the sclerostomy. The tube was then affixed to the sclera and covered with a Tutoplast scleral patch graft. Overlying Tenon and conjunctival tissues were re-approximated and closed.

For the Baerveldt placement, a conjunctival periotomy was created. A Baerveldt-250 or Baervedlt-350 implant was placed either in the superonasal or superotemporal quadrants below the rectus muscles and anchored to the sclera with two 8-0 nylon sutures. The tube was then ligated with an absorbable 6-0 vicryl suture for hypotony prevention. When tube fenestration was performed, this was done with the needle of the 6-0 vicryl suture. The tube was trimmed to an appropriate length with a beveled tip. A sclerostomy was made, and the tube is inserted into the anterior chamber through the sclerostomy. The tube was then affixed to the sclera and covered with a Tutoplast scleral patch graft. Overlying Tenon and conjunctival tissues were re-approximated and closed.

Data were collected at baseline visits prior to surgery and at 6 and 12 months after surgery. Baseline demographic and ocular characteristics such as sex, age, medical comorbidities, uveitis etiology and location, and other ophthalmic history were collected. We also recorded preoperative and postoperative IOP, best-corrected visual acuity, topical and systemic glaucoma medications, systemic immunosuppressant use, postoperative complications, and reoperations. Postoperative complications included hypotony, choroidal effusion, cystoid macular edema, corneal edema/decompensation, and severe inflammation. Complications were divided into early complications (6 weeks to 4 months after surgery) and late complications (> 4 months after surgery).

The primary outcome measures were IOP reduction and failure rate. Failure was defined as one of the following: IOP out of target range (5–21 mmHg), < 20% reduction from baseline at two consecutive follow-up visits after 3 months, glaucoma reoperation, or loss of light perception [17].

Statistical analysis was performed using SAS 9.4 (SAS Institute Inc., Cary, NC, USA). Snellen visual acuity measurements were converted to logMAR equivalents. Descriptive statistics were performed to compare baseline demographic and ocular characteristics among treatment groups. Continuous variables were analyzed using the Kruskal-Wallis test, and categorical variables were analyzed using the Pearson’s chi-squared test and Fisher’s exact test.

The Pearson chi-squared test, Fisher’s exact test, and Kruskal-Wallis test were performed to identify potential risk factors for failure. Logistic regression was used to determine the relative risk of failure among treatment groups. Time to failure was estimated using Kaplan-Meier curves and compared using the Wilcoxon test.

Statistical significance was defined as *p* < 0.05.

## Abbreviations

ABC: Ahmed Baerveldt Comparison Study
AVB: Ahmed Versus Baerveldt Study
Glc: Glaucoma
HLA: Human leukocyte antigen
IOP: Intraocular pressure
logMAR: Logarithm of minimum angle of resolution
MMC: Mitomycin C
TVT: Trabeculectomy Versus Tube Study
USC: University of Southern California
